# Lessons for non-VA care delivery systems from the U.S. Department of Veterans Affairs Quality Enhancement Research Initiative: QUERI Series

**DOI:** 10.1186/1748-5908-4-9

**Published:** 2009-02-26

**Authors:** Leif Solberg

**Affiliations:** 1 HealthPartners Medical Group and HealthPartners Research Foundation, Minneapolis MN, USA

## Abstract

The U.S. Veterans Health Administration (VHA) may have a very different structure and function from the organizations and practices that provide medical care to most Americans, but those organizations and practices could learn a lot from the VHA's Quality Enhancement Research Initiative (QUERI). There are at least six topics of increasing importance for implementation research where QUERI experience should be of value to other non-VHA organizations, both within and external to the United States: 1) Researcher-clinical leader partnerships for care improvement; 2) Attention to culture, capacity, leadership, and a supportive infrastructure; 3) Practical economic evaluation of quality implementation efforts; 4) Human subject protection problems; 5) Sustainability of improvements; and 6) Scale-up and spread of improvements.

The articles in *Implementation Science's *QUERI Series provide the details of those lessons for others who are willing to invest the time to translate them into their different settings.

## Background

The initial reaction of most American care delivery leaders to the question of what they can learn from the U.S. Department of Veterans Affairs Quality Enhancement Research Initiative (QUERI) and the transformation of care quality in the Veteran's Health Administration (VHA) would probably be – very little. Most would see the VHA health care system as completely different from care for most Americans: huge, national, centralized, traditionally focused on inpatient care, managed through a government bureaucracy with limited flexibility, unconcerned about competition or costs, and caring for an atypical group of mostly poor elderly male patients with limited ability to go elsewhere for care. In particular, what could the relatively small medical practices that provide over 80% of the medical care in this country learn from this seemingly irrelevant giant care system?

After getting to know the VHA and QUERI through various QUERI leaders as well as through the papers included in *Implementation Science's *QUERI Series, I am convinced that such preliminary impressions are understandable but wrong. Few should expect to simply duplicate the VHA approach to quality improvement, which clearly operates in relatively arcane, bureaucratic, and acronym-encumbered ways. However, any healthcare delivery system that can so dramatically improve its care quality in a surprisingly short time must have lessons for the rest of us [1].

The remarkable quality achievements of the VHA system were objectively demonstrated by Asch and colleagues in their use of a variety of performance measures to compare 12 community samples with 12 VA systems [2]. Patients from the VHA scored significantly higher for adjusted overall quality (67% vs. 51%), chronic disease care (72% vs. 59%), and preventive care (64% vs. 44%), but not for acute care. Moreover, the "differences were greatest in areas where the VHA has established performance measures and actively monitors performance." From the standpoint of an external medical group willing to learn from this experience, there are at least two important questions. First, to what extent have the quality improvements been due to QUERI versus other structural and process changes implemented by VHA leadership? Other organizations need to know both whether they must develop similar research and operational partnerships for best results and what management changes might be most beneficial.

One hint that QUERI may not be completely responsible for the improvements comes from knowing that the organizational changes began in 1995, and had already accomplished much by the time that QUERI could have had much effect by 2000 [1,3]. These changes included: making it a primary organizational priority to produce dramatic quality improvements, reorganizing care delivery into 22 regions or VISNs (Veterans Integrated Service Networks), establishing quantitative performance measurement metrics and using them to monitor comparisons and accountability, aligning resource allocation with quality goals, adopting system-wide practice guidelines, and implementing electronic records and information systems. Other answers could come from comparing performance improvement for the eight topic areas created by QUERI with others not so targeted but still part of the VHA performance measurement set. So far as I can tell from either the articles in the QUERI Series or those published elsewhere, this has not been done. Thus, while it is very tempting to believe that QUERI must have been important in the VHA improvements for someone who firmly believes that integrating research and practice is key to real quality improvement in health care, none of the articles in this Series prove that.

The other important question is which of the lessons from QUERI do not depend on the unique characteristics of the VHA? For example, lessons that should be applicable in other care systems within or beyond the U.S. and medical practices regardless of size. I have identified six important issues where others should be able to take advantage of the QUERI approach, even though they may not want, need, or be able to adopt the QUERI model: 1) Researcher-clinical leader partnerships for care improvement; 2) Attention to culture, capacity, leadership, and a supportive infrastructure; 3) Practical economic evaluation of quality implementation efforts; 4) Human subject protection problems; 5) Sustainability of improvements; and 6) Scale-up and spread of improvements.

## Discussion

### Researcher-clinical leader partnerships

The first and most important issue addresses the large gap or chasm separating what we think we know from what we do in healthcare delivery, as highlighted by the Institute of Medicine in its landmark 2001 report [4]. Van de Ven and Johnson have noted that this large gap between theory and practice is not unique to medicine, but is common in most professions and businesses today [5]. They discuss three alternative ways of viewing this gap.

1. Practice knowledge comes from research, so it is a knowledge transfer problem. (It is usually described in medicine as a problem of translation.)

2. Practice and research knowledge are different in nature, each distinct and complementary, but not directly transferable to each other.

3. Production of useful knowledge is the real problem, which requires what they call "engaged scholarship" and research-practice partnerships.

In their view, the third concept is correct, and there are very few examples of such partnerships in which the agenda (research questions) comes from practice, and both parties participate in developing, conducting and implementing lessons from the research. That makes the lessons from QUERI invaluable for other settings. Smith et al describe how the Mental Health QUERI used partnerships to develop and evaluate the spread of evidence-based collaborative care for depression [6]. While they describe the approach being used that relies on participation and feedback by both researchers and VHA operational leaders, the formative evaluation hasn't been completed, so it is too early to learn what worked and what didn't. However, they observe that this kind of partnership creates a problem for health services researchers by requiring a new role of them. Although new funding mechanisms and organizational structures may be required to permit this approach to thrive, we shall have to await completion of the project to know what generalizable lessons it produces.

In our large integrated care delivery system, we have independently learned the importance of such research-practice partnerships, especially if the goal is practice improvement rather than a one-way implementation of research evidence. We have explored this approach with other care delivery leaders elsewhere and find rather widespread agreement with the need, although all of us are still learning how to do it well [7]. Reading between the lines of QUERI description articles can help those working on building partnerships.

### Pay attention to culture, capacity, leadership, and a supportive infrastructure

Stetler et al describe the framework and strategies used to implement the QUERI approach to research-based practice [8]. They point out some of the main barriers and facilitators that QUERI backers in the VHA had to address in its implementation. Chief among these were three interacting and overlapping elements for organizational change that required leadership with clear expectations. These also will need attention in any care setting: culture change for both researchers and clinical care leaders; provision of capacity, capability, and resources; and supportive infrastructures to reinforce and sustain new behaviors

In our study of what is needed for transforming care in large multi-specialty group practices, we identified similar elements [9]. We also found those elements to be equally important in a case study of an especially successful small primary care practice [10]. A relatively simple conceptual framework built on those and other research and organizational change experiences in a wide variety of medical practices may be easier for non-VHA practice leaders to understand and use than the complex QUERI organizational framework, but the principles are very similar [11].

### Practical economic evaluation of implementation

In the rest of the health care system, there is great concern about the financial implications of any change, even if it does result in improved quality. This problem becomes especially complicated because current payment systems often create misaligned incentives that benefit some parties while hurting others. Current research approaches to cost-effectiveness analysis are rarely used in decision-making for many reasons [12]. Not least of these reasons is the requirement by The Panel on Cost-Effectiveness in Health and Medicine to focus on societal costs rather than those of the parties who must charge and pay for the care [13]. QUERI economists Smith and Barnett have made an important contribution to our ability to understand the actual costs of medical business decisions by highlighting business case analysis (BCA) and contrasting it with the traditional cost effectiveness analysis (CEA) used by researchers [14]. BCA has the advantage of separating the costs of delivery from those of implementation, even separating the latter into the costs of initial engagement, direct implementation, and indirect effects on health care utilization. Most importantly, BCA takes the provider's perspective, so it only counts those costs incurred by the provider, and it does so over a short time horizon of 1–5 years rather than lifetime. Apparently this approach is starting to be used widely in QUERI studies, including using it as a tool to engage managers in the spread process. While no actual examples are provided in the Smith report, future reports on specific quality improvement efforts using the BCA methodology should allow other large care systems to understand the cost implications for them.

### Human subject protection problems

There is already considerable concern and confusion about whether and how quality improvement (QI) projects should be required to undergo formal review for human subjects protection [15]. This is a particular problem when the results of a QI project warrant publication for the benefit of other organizations, and the journal editor requires a statement confirming that the project had institutional review board (IRB) approval. Since the current IRB system requires prospective review, this catch-22 can prevent publication and actually damage the best interests of patients. These problems and the time, effort, and confusion caused by needing to work with multiple IRBs for multi-site studies are clearly issues for QUERI, as described by Chaney et al [16]. They describe quantitatively how their depression improvement evaluation required 160 reports to multiple IRBs with varying approaches and long time delays. If they succeed in facilitating movement toward centralized IRBs, a specific IRB structure for QI projects, and clarification of guidelines for implementation and QI research, it will be a very important model for the rest of the world.

### Sustainability of improvements

Leaders of large healthcare delivery organizations and small practices alike know too well that sustaining successful improvements is at least as hard as implementing them in the first place. However, research studies rarely either study this process or report on what happens after the research team goes away, in part because of their lack of interest in this problem and in part because of the constraints of funding. While the report of Bowman et al suggests that the funding problem for QUERI evaluations is as important in the VHA as elsewhere, the organizational priority for maintaining change seems likely to produce information about this problem [17]. They describe a supplemental study of a QUERI program to improve HIV/Hepatitis care that developed measures of continued use of new care processes and outcomes. They found it important to distinguish between maintenance failures caused by external influences versus internal lack of perceived utility. The most important lesson of their study was the need to have measures of sustainability built into the original project rather than as a post hoc add-on. They recommend that research funders require sustainability analyses and note that was part of a 2005 VHA project solicitation.

While funding support for more complex sustainability measures may not be likely or seem relevant for small or large practices doing their own quality improvement initiatives, the concept is still important. Any size group can identify a few key process or outcome measures of desired improvements that are simple enough to be repeated periodically and monitored on a time chart. Learning to rely on such data is an important part of becoming a learning organization, just like QUERI has helped the VHA to become [18].

### Dissemination and spread of improvements

A related aspect of improvement implementation that is usually neglected in the current literature is scale-up and spread of successful projects. Again, the national scope of the VHA makes it very important to address and solve this issue, but it is also a problem for most multi-site care systems. In an ongoing QUERI study, Luck and colleagues examine the Mental Health QUERI Center's use of a social marketing approach to reach the various audiences important to spread: national and VISN leaders, facility managers, clinicians, and veterans. Although their work is an effort in progress, its delineation of strategies for different target audiences may be useful for others. Berwick has described this scale-up/spread problem, identifying seven recommendations for accelerating the diffusion of innovations, and they sound a lot like the lessons from QUERI [19].

## Summary

Although the administrative and scope characteristics of the VHA make many of the specific answers to the issues described above irrelevant to even large healthcare delivery organizations in the U.S., the issues themselves and generic lessons from QUERI are important for any size care system or clinic anywhere. That importance and its relevance will be clearer if QUERI authors publish their findings in language and styles that are equally relevant to those audiences. That has not been particularly true of most of the articles in this Series, which seem primarily aimed at those within the VHA and QUERI. Some previous articles have provided better examples of such an approach, especially those of Rubenstein and Pugh for implementation researchers and of Hagedorn et al for anyone implementing research evidence into clinical practice [20,21]. However, QUERI has much to teach those who are willing to work at translating its now extensively published lessons into usable ideas for their settings, no matter how different those settings may seem to be.

## Competing interests

The author declares that they have no competing interests.
